# Efficacy of salvage therapies after failure of adjuvant anti-PD-1 monotherapy for melanoma in the Chinese population: a multi-institutional cohort study

**DOI:** 10.1007/s10637-023-01348-5

**Published:** 2023-04-24

**Authors:** Dong-Dong Jia, Yu Xu, Ting Li, Ji-Long Yang, Yong Chen, Tao Li

**Affiliations:** 1 Department of Bone and Soft-tissue Surgery, The Cancer Hospital of the University of Chinese Academy of Sciences (Zhejiang Cancer Hospital), Institute of Basic Medicine and Cancer (IBMC), Chinese Academy of Sciences, Hangzhou, Zhejiang 310022 China; 2 Department of Musculoskeletal Surgery, Department of Oncology, Fudan University Shanghai Cancer Center, Shanghai Medical College, Fudan University, Shanghai, 200032 China; 3 Departments of Bone and Soft Tissue Tumour, Tianjin Medical University Cancer Institute and Hospital, Tianjin, 300060 China

**Keywords:** Melanoma, Salvage therapies, Adjuvant monotherapy, Chinese population

## Abstract

The majority of melanoma patients experience relapse during adjuvant therapy or after the end of therapy. Sixty-one patients from 3 melanoma centres who experienced recurrence and received adjuvant pembrolizumab for resected stage III/IV melanoma were enrolled. Disease characteristics, recurrence characteristics, subsequent management and outcomes were retrospectively analysed. Sixty-one patients were enrolled in this study. The median time to first relapse from the commencement of adjuvant pembrolizumab was 8 months (1–22 months). The first recurrences were locoregional alone in 25 patients (41%), distant alone in 29 (47.5%) and concurrent locoregional and distant relapse in 7 (11.5%). At the first recurrence, 4 patients (80%) who underwent resection alone experienced further relapse of disease. Three (60%) patients who were treated with adjuvant pembrolizumab following surgery, 2 (100%) patients who were treated with adjuvant chemotherapy, 2 (66.7%) patients who were treated with adjuvant chemotherapy and pembrolizumab combined and 3 (100%) patients who were treated with adjuvant radiotherapy and pembrolizumab combined had further recurrence. Of the three patients treated with adjuvant BRAF/MEKi following the first relapse, none had yet recurred. Of the 8 patients treated with pembrolizumab alone, only one patient (12.5%) who recurred after ceasing adjuvant PD1 had a partial response. The overall response rate to BRAF/MEKi was 75%, 3/4; to pembrolizumab in combination with an oral multitargeted receptor tyrosine kinase inhibitor, it was 22.2%, 2/9; to chemotherapeutic agents alone, it was 33.3%, 1/3; and to chemotherapeutic agents combined with pembrolizumab, it was 37.5%, 3/8. The patient treated with imatinib had progressive disease after 3 months of treatment. Of the 6 patients who received temozolomide combined with pembrolizumab, 3 (3/6, 50%) had a partial response. The median OS of the patients who relapsed locoregionally only was longer than that of the patients who relapsed distally at the first recurrence (35 months and 14 months, respectively; P < 0.01). The outcomes of the patients with disease recurrence during or after the completion of 1 year of adjuvant anti-PD1 therapy were poor despite multimodality treatment.

## Introduction

Malignant melanoma is the deadliest cutaneous malignancy, with approximately 90,000 new cases diagnosed annually in America and 20,000 new cases in China [1]. Immune checkpoint inhibitors, especially anti-PD-1 agents, have dramatically changed the landscape of the treatment of advanced melanoma.

Pembrolizumab, an anti-PD-1 agent, is a highly selective humanised IgG4 monoclonal antibody that blocks the interaction between PD-1 (programmed cell death 1) on T cells and its ligands, PD-L1 and PD-L2 (programmed cell death ligand 2), to functionally hamper the immune escape of tumour cells [2, 3]. Based on the positive oncological outcomes from the KEYNOTE-054 trial, pembrolizumab was approved for the treatment of resected stage III and IV melanoma in the adjuvant setting in 2019 in the United States and China [4]. Nevertheless, adjuvant anti-PD-1 monotherapy leads to a limited response in resected stage III melanoma cases among Asian populations [5]. The majority of melanoma patients experience relapse during adjuvant therapy or after the end of therapy.

Hence, how to proceed if relapse or metastasis occurs during or after adjuvant treatment remains a question. Identification of the optimal treatment for melanoma that has progressed on adjuvant anti-PD-1 therapy is a large unmet need. Although the potential benefits of management after adjuvant anti-PD-1 failure are being validated in Caucasian populations, little is known in Asian populations. The aim of this study was to describe patterns of relapse, management and outcomes in Chinese patients with melanoma after adjuvant PD-1 failure.

## Methods

Data were extracted retrospectively from three cancer centres in China. Between May 2018 and May 2020, all completely resected stage III or IV melanoma patients with no evidence of disease who received at least one dose of adjuvant pembrolizumab and experienced recurrence were enrolled. Whether wide surgical margins were required for primary melanoma were determined according to the suggestions in the NCCN Guidelines. All patients with lymph node metastases received standard complete lymph node dissection. The disease characteristics before adjuvant therapy, such as primary site, AJCC 8th edition staging and type of mutation, were collected. Details on adjuvant anti-PD-1 antibody therapy were noted, including drug dosage, duration and reason for the cessation of treatment. Information was recorded on sites of recurrent disease, subsequent management and outcomes. Imaging type and frequency were determined according to the standard of care at each cancer centre. Clinical outcomes were estimated by investigator review: local skin, in-transit or nodal metastases were deemed locoregional recurrence, any distant or visceral metastases were deemed distant recurrence, any degree of tumour regression was reported as partial response, and overall survival (OS) data were reported from surgery or first line of systemic therapy at relapse.

The Kaplan‒Meier method was used to estimate OS. All statistical analyses were performed using SPSS version 24.0 (IBM Corp, Armonk, NY, USA). Differences with a p value < 0.05 (two-sided) were considered statistically significant.

## Result

### Patient characteristics and adjuvant therapy

A total of sixty-one patients were enrolled in this study. The patients’ baseline demographic characteristics are summarised in Table 1. Of these patients, 50.8% (n = 31) were from Zhejiang Cancer Hospital, with the remainder from Fudan University Shanghai Cancer Center (n = 16, 26.2%) or Tianjin Cancer Hospital (n = 14, 23.0%). The median age at diagnosis was 61 years (range 39–77), with a slight male predominance of 52.5% (n = 32). Thirty-seven patients (60.7%) had acral melanoma and 21 patients (34.4) had nonacral cutaneous melanoma, with three (4.9%) having no identifiable skin lesion. A total of 18.0% (n = 11) of the patients had a confirmed BRAF V600 mutation, while 6.6% (n = 4) had mutations and/or gene copy number increases in c-Kit. According to the 8th edition of the AJCC staging system, 5/15/29/7 patients had a diagnosis of stage IIIA/IIIB/IIIC/IIID disease, and 5 subjects (8.2%) had resected stage IV disease. Forty-six (75.4%) patients underwent completion lymph node dissection surgery prior to adjuvant therapy.

**Table 1:** Baseline patient characteristics

Characteristics	Patient number, N = 61 (%)
Sex–no. (%)	
Male	32 (52.5)
Female	29 (47.5)
Median age at diagnosis (range)	61 (39–77)
Primary Site–no. (%)	
Cutaneous	21 (34.4)
Acral	37 (60.7)
Occult	3 (4.9)
BRAF mutation–no. (%)	
Mutation	11 (18.0)
Wild	50 (82.0)
Patient origin–no. (%)	
Zhejiang	31 (50.8)
Shagnhai	16 (26.2)
Tianjin	14 (23.0)
Melanoma Stage at Adjuvant Treatment (AJCC 8th edition)–no. (%)	
Stage III	56 (91.8)
IIIA	5 (8.2)
IIIB	15 (24.6)
IIIC	29 (47.5)
IIID	7 (11.5)
Resected stage IV	5 (8.2)

Forty-two patients (68.9%) with lower body weight received adjuvant low-dose pembrolizumab 100 mg every 3 weeks, and 19 patients (31.1%) with a higher body weight received adjuvant high-dose pembrolizumab 200 mg every 3 weeks. Four (6.5%) patients received adjuvant radiotherapy (RT) prior to first recurrence. The median duration of adjuvant immunotherapy was 8 months. A total of 29.5% of the patients ceased adjuvant immunotherapy owing to completion of the prescribed course.

### Disease characteristics at the initial recurrence

The median time to first relapse from the commencement of adjuvant pembrolizumab was 8 months (1–22 months). Forty-three (70.5%) patients experienced recurrence during adjuvant immunotherapy, and 18 (29.5%) patients experienced recurrence after the cessation of adjuvant immunotherapy. The first recurrences were locoregional alone in 25 patients (41%), distant alone in 29 (47.5%) and concurrent regional lymph nodes and distant relapse in 7 (11.5%). Of the 25 patients who relapsed locoregionally, 3 patients relapsed in the primary sites, 8 patients developed in-transit metastases, 12 relapsed in the regional lymph nodes, and 2 patients developed both in-transit and regional lymph node metastases.

### Treatment at first recurrence and response to 1st line therapy

At first recurrence, 5 (8.2%) patients underwent resection alone, of whom 4 (80%) experienced further relapse of disease. Several patients with resectable recurrence underwent surgery, either with adjuvant pembrolizumab (5, 8.2%), adjuvant chemotherapy (2, 3.3%), adjuvant chemotherapy and pembrolizumab combined (3, 4.9%), adjuvant radiotherapy and pembrolizumab combined (3, 4.9%) or adjuvant BRAF/MEKi (3, 4.9%).

Three (60%) patients who were treated with adjuvant pembrolizumab following surgery, 2 (100%) patients who were treated with adjuvant chemotherapy, 2 (66.7%) patients who were treated with adjuvant chemotherapy and pembrolizumab combined and 3 (100%) patients who were treated with adjuvant radiotherapy and pembrolizumab combined had further recurrence. Of the three patients treated with adjuvant BRAF/MEKi following the first relapse, none had yet recurred.

Of the 32 patients who received systemic therapy, 8 (25%) had pembrolizumab alone, 4 (12.5%) had BRAF/MEKi, 9 (28.1%) had pembrolizumab in combination with an oral multitargeted receptor tyrosine kinase inhibitor, 11 (34.4%) had chemotherapeutic agents alone or combined with pembrolizumab (3 had monotherapy, 8 had combination with PD1) and 1 (3.1%) had imatinib alone.

**Fig. 1:**
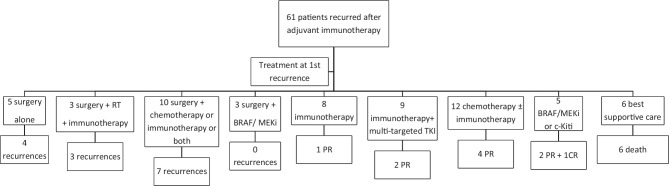
Flow chart of treatment at first recurrence of melanoma after adjuvant immunotherapy. RT radiotherapy, PR partial response, CR complete response

Of the 8 patients treated with pembrolizumab alone, only one patient (12.5%) who recurred after ceasing adjuvant PD1 had a partial response (7 progressive disease). The overall response rate to BRAF/MEKi was 75%, 3/4 (one complete response), to pembrolizumab in combination with an oral multitargeted receptor tyrosine kinase inhibitor, it was 22.2%, 2/9 (0 complete responses), to chemotherapeutic agents alone, it was 33.3%, 1/3 (0 complete responses) and to chemotherapeutic agents combined with pembrolizumab, it was 37.5%, 3/8 (0 complete responses). The patient treated with imatinib had progressive disease after 3 months of treatment. Of the 6 patients who received temozolomide combined with pembrolizumab, 3 (3/6, 50%) had a partial response (Fig. 1).

### Overall survival

**Fig. 2:**
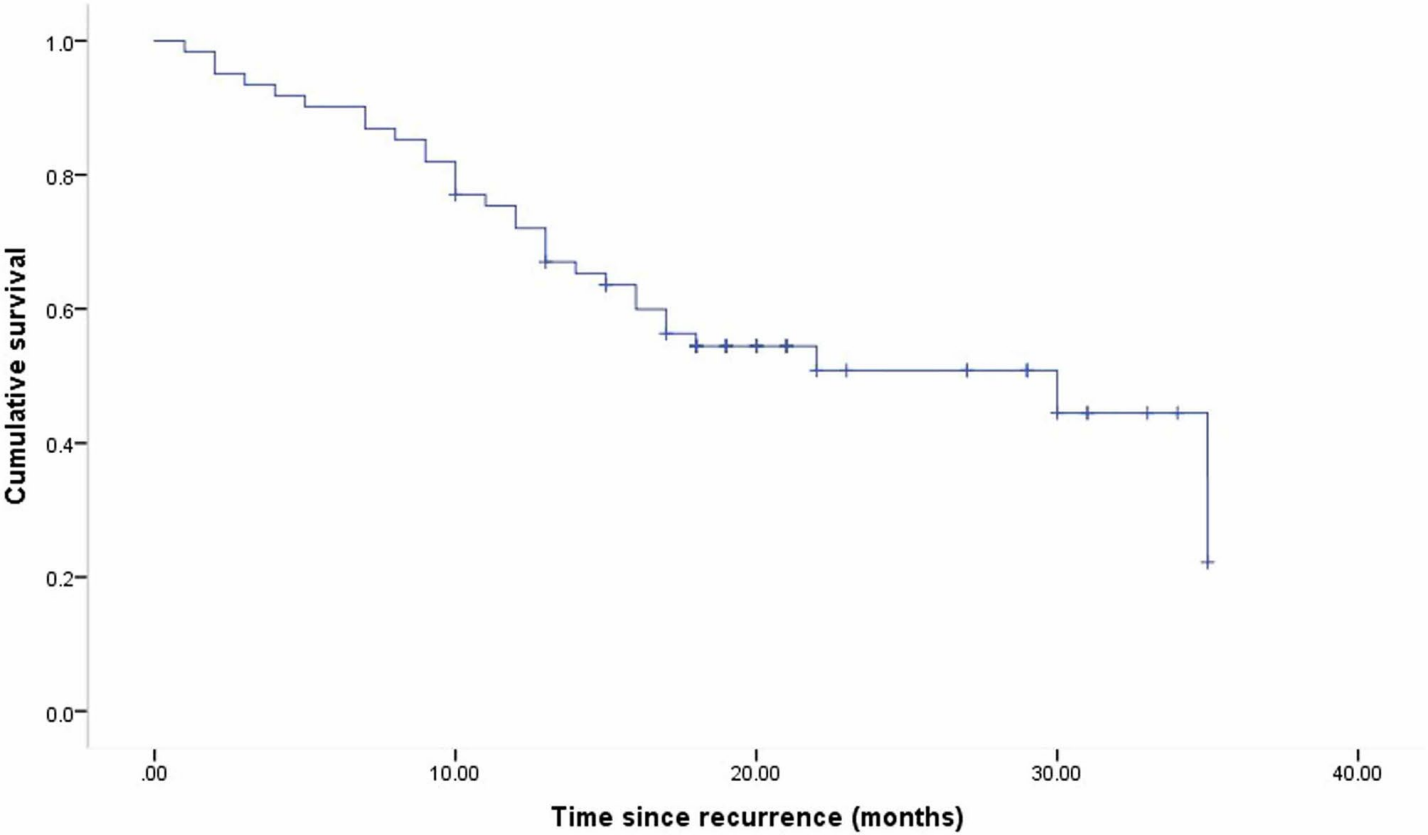
Kaplan–Meier curve of overall survival for all patients from the time of first melanoma recurrence

**Fig. 3:**
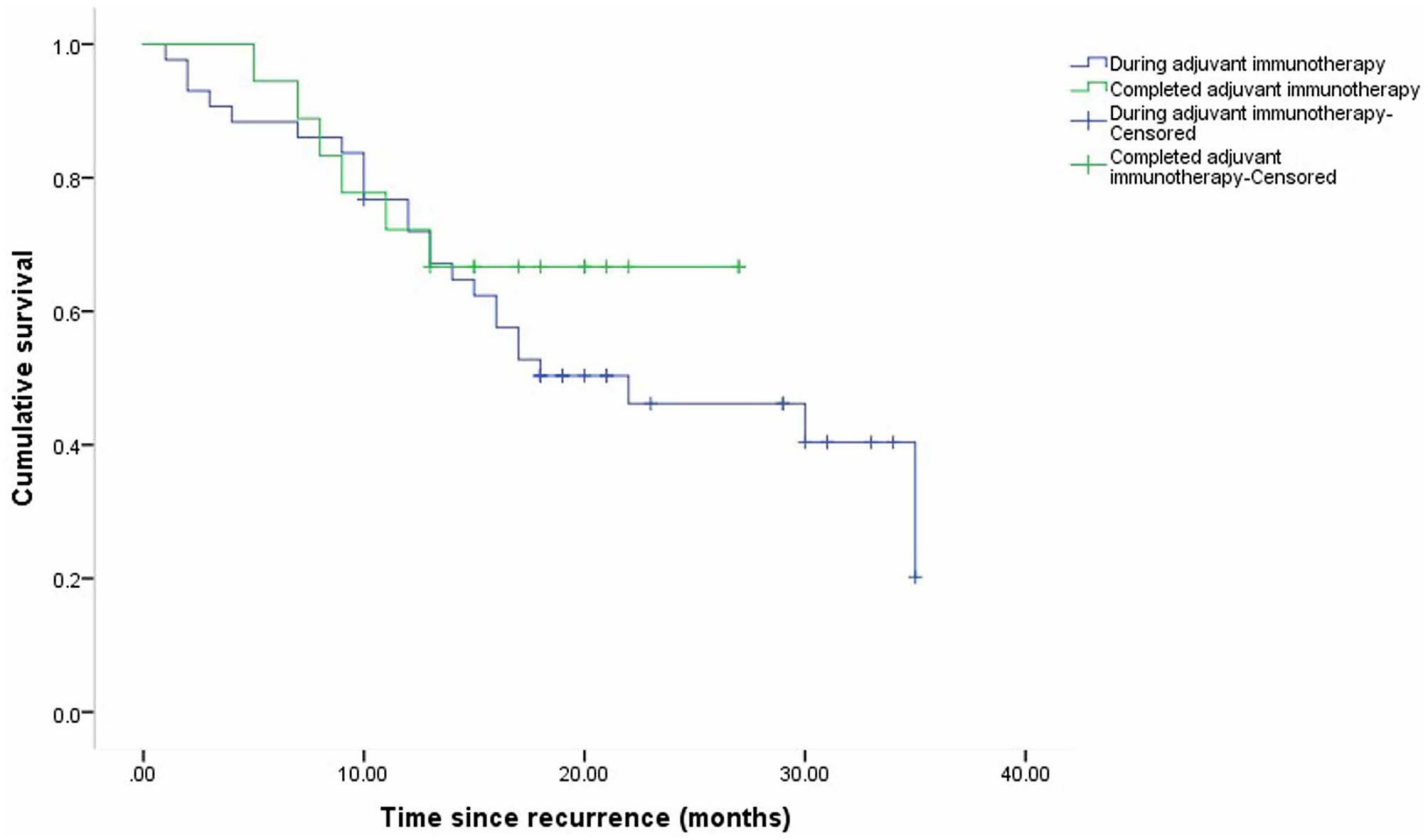
Kaplan–Meier curve of overall survival for patients who relapsed during and after adjuvant immunotherapy (p = 0.38)

**Fig. 4:**
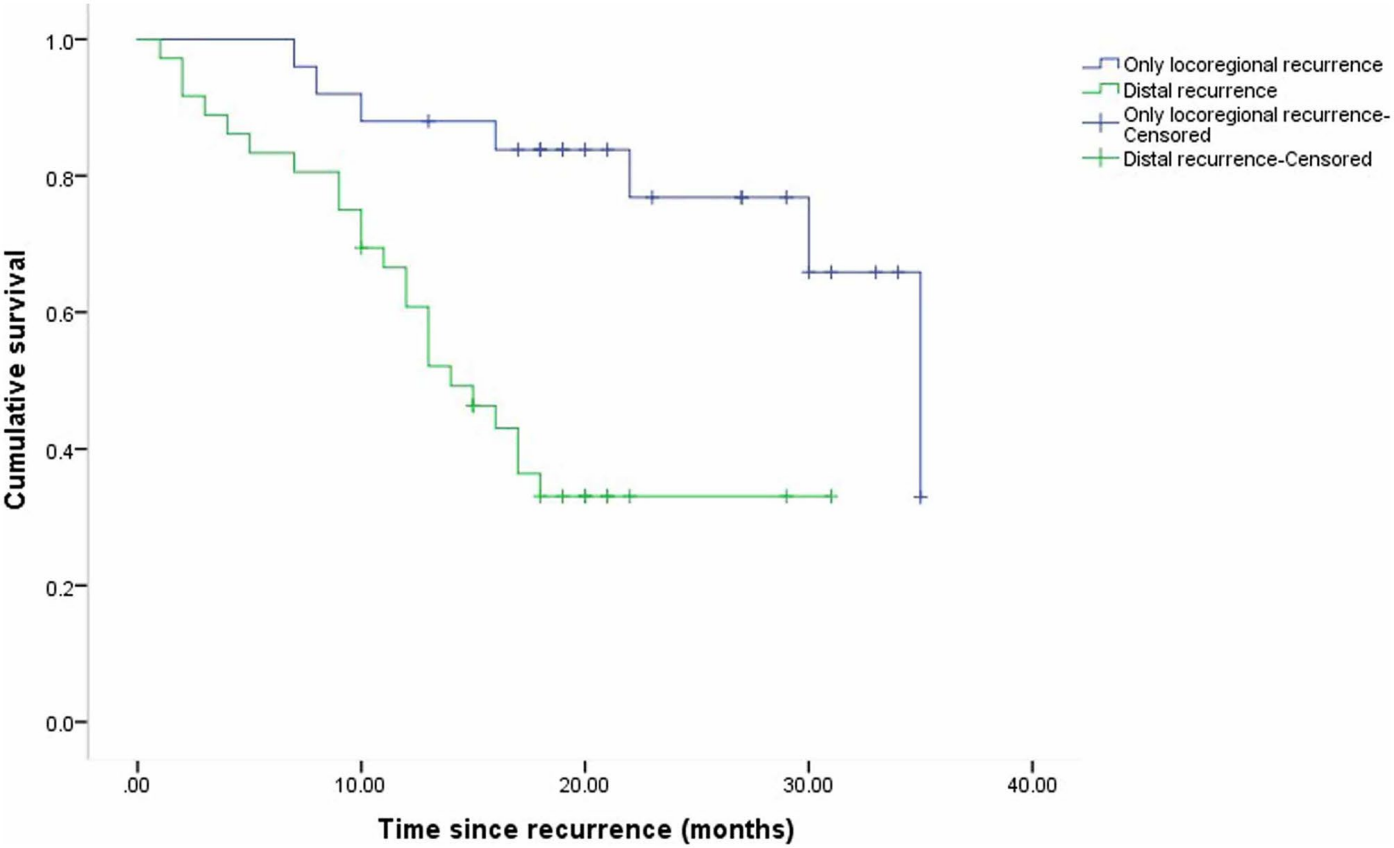
Kaplan–Meier curve of overall survival for patients who relapsed locoregionally only and distally at first recurrence (P < 0.01)

The median overall survival from the date of the initial relapse for all the enrolled patients was 30 months (95% CI 11.63–48.37) (Fig. 2). There was no significant difference in OS between the patients who relapsed before or after ceasing adjuvant immunotherapy (p = 0.38) (Fig. 3). The median OS of the patients who relapsed locoregionally only was longer than that of the patients who relapsed distally at the first recurrence (35 months and 14 months, respectively; P < 0.01) (Fig. 4).

## Discussion

Adjuvant immunotherapy has significantly reduced the chance of recurrence. The Keynote-054 trial revealed that adjuvant pembrolizumab led to a 20% improvement in RFS compared with placebo [4]. However, due to the lack of a deep understanding of PD-1 blockade resistance mechanisms, mechanisms of overcoming resistance and markers for monitoring antitumour activity, some of the patients suffer from recurrent disease during adjuvant therapy or after the end of therapy. The treatment selection of patients who relapse during or after adjuvant immunotherapy is unanswered and includes treatment switch at recurrence, response rates and survival benefit. Owing to a lack of available data, there is no solution other than to follow clinical experience or enrol the patient in a clinical trial after adjuvant therapy failure.

In our study, 59% of the recurrences were distant with or without locoregional relapse. It revealed that pembrolizumab is a more efficient adjuvant therapy in decreasing distant metastases than in decreasing locoregional recurrence, which was consistent with data from the KEYNOTE-054 [6] study. Favourable overall survival may be associated with locoregional recurrence only rather than distant metastases.

A preliminary retrospective analysis was performed to study the patterns of recurrence and salvage therapies following adjuvant anti-PD1 therapy. In this study, 70.5% of the enrolled patients had melanoma recurrence during adjuvant anti-PD1 therapy, and 29.5% of the patients recurred following treatment cessation. The median time to relapse from the commencement of adjuvant therapy was longer than that in a retrospective study conducted by Owen et al. (8 months vs. 4.6 months) [7].

The primary considerations for retreatment after adjuvant PD-1 failure include site of relapse, chronology of adjuvant immunotherapy and relapse, BRAF mutation status, performance status, wishes and financial status of the patient.

The patients who underwent resection alone after the first recurrence had a high relapse rate of 80%. These data suggest that surgery alone in the setting of melanoma relapse after adjuvant immunotherapy may not result in long-term disease control and that adjuvant systemic therapy is needed. It should be noted that no relapses were observed in the three patients treated with adjuvant BRAF/MEKi after resection.

A case report revealed that ipilimumab might be an effective treatment choice after adjuvant immunotherapy failure in a patient with stage IIIC malignant melanoma [8]. A reported phase 2 trial of pembrolizumab combined with ipilimumab resulted in an RR of 29% in metastatic melanoma patients who failed PD-1/L1 therapy [9]. However, a multi-institutional historical cohort study suggested that salvage nivolumab plus ipilimumab and ipilimumab monotherapy showed limited efficacy in Japanese populations with advanced melanoma after PD1 resistance. Nevertheless, ipilimumab is not licenced for the treatment of melanoma in China, and ipilimumab cannot be evaluated as a potential promising regimen.

Owen et al. demonstrated that 40% of the patients who recurred after cessation of adjuvant immunotherapy responded to a rechallenge of anti-PD-1 therapy [7], but 12.5% of the patients in the same setting experienced a response in our study. Of note, only five and eight patients were enrolled in this cohort of two studies. In addition, anti-PD1 agent rechallenge may be an available alternative for patients who have ceased adjuvant immunotherapy.

To evaluate the combination of lenvatinib and pembrolizumab in patients with advanced melanoma with confirmed progression on a PD-1/L1 inhibitor, the LEAP-004 study (NCT03776136) was performed. The overall ORR was 21.4% (95% CI 13.9–30.5), the DCR was 65.0%, and the median DOR was 6.3 months. This phase 2 study revealed that lenvatinib plus pembrolizumab had activity in this population. Similarly, pembrolizumab in combination with an oral multitargeted receptor tyrosine kinase inhibitor may also have activity in selected patients, with a response rate of 22.2% in our study.

In a retrospective study, an overall response rate of 82% was observed in melanoma patients treated with BRAF/MEKi after PD1 failure [7]. BRAF/MEKi also led to a favourable response for BRAF-mutated melanoma in a Japanese population who relapsed after PD1 failure. In this series, our data showed a similar trend, in which patients treated with BRAF/MEKi had an ORR of 75% (3/4).

For advanced melanoma patients harbouring c-Kit mutations or amplifications, imatinib mesylate is an alternative therapy with a favourable response rate of 23.3% [10]. In this study, no patient (0/1) responded to imatinib mesylate.

Chemotherapeutic agents were deemed to have immunomodulatory effects, such as enhancement of tumour immunogenicity and disruption of immune-suppressive pathways [11–13]. Chemotherapy drugs and anti-PD-1 agents showed synergistic antitumour activity in non-small cell lung cancer (NSCLC), melanoma and other cancers [14, 15]. A retrospective study demonstrated a superior event-free survival (EFS) benefit (median EFS of 7.6 months in combination regimens vs. 3.4 months in monotherapies) and ORR benefit (ORR 59% in combination regimens vs. 15% in monotherapies) of chemotherapy drugs combined with anti-PD-1 agents compared with monotherapy of chemotherapy drugs or immune checkpoint inhibitors.

In a retrospective study conducted by Fudan University Shanghai Cancer Center, an ORR of 40% was reached in advanced melanoma patients treated with a combination of pembrolizumab with temozolomide as first-line therapy, and 55% of them achieved a durable response [16]. We demonstrated that temozolomide plus pembrolizumab can induce clinical responses in patients whose disease progressed after adjuvant anti-PD-1 monotherapy, resulting in a favourable ORR of 50%. The high ORR and short time-to-response in the temozolomide plus pembrolizumab group suggested that this combination can be used to achieve rapid disease debulking. It is worth noting that the 6 patients treated with temozolomide plus pembrolizumab were from Fudan University Shanghai Cancer Center.

In melanoma patients whose diseases progressed during or after adjuvant pembrolizumab, a higher objective response (approximately 26%) to subsequent chemotherapies was observed [15]. In this study, we demonstrated an improved ORR of the combination of carboplatin and paclitaxel compared with that of chemotherapy historic controls in the preimmunotherapy era [17].

Additionally, we revealed that the combination of temozolomide and pembrolizumab after adjuvant anti-PD-1 therapy failure is superior to chemotherapy in the same setting.

From these data, the options for first disease recurrence occurring during adjuvant anti-PD1 monotherapy or after completion of 1-year adjuvant anti-PD1 agents include surgery with adjuvant therapy, BRAF/MEK inhibitors (for BRAF mutant patients), pembrolizumab in combination with an oral multitargeted receptor tyrosine kinase inhibitor, and chemotherapeutic agents alone or with pembrolizumab. Rechallenge with a PD-1 inhibitor is available for patients who relapse after the completion of adjuvant PD-1 inhibition.

Despite including three large cancer centres, we could only recruit a modest number of patients. The follow-up time and imaging methods were not stringently regulated, and a centralised review was not performed. Due to these limitations, our results need to be further validated using a prospective study with a large sample size to minimise the heterogeneity in the population.
